# Auditory Tones and Foot-Shock Recapitulate Spontaneous Sub-Threshold Activity in Basolateral Amygdala Principal Neurons and Interneurons

**DOI:** 10.1371/journal.pone.0155192

**Published:** 2016-05-12

**Authors:** François Windels, Shanzhi Yan, Peter G. Stratton, Robert Sullivan, James W. Crane, Pankaj Sah

**Affiliations:** 1 Queensland Brain Institute, The University of Queensland, Brisbane, Queensland, Australia; 2 Asia Pacific Centre for Neuromodulation, Queensland Brain Institute, Brisbane, Queensland, Australia; 3 School of Biomedical Sciences, Charles Sturt University, Bathurst, New South Wales, Australia; McGill University, CANADA

## Abstract

In quiescent states such as anesthesia and slow wave sleep, cortical networks show slow rhythmic synchronized activity. In sensory cortices this rhythmic activity shows a stereotypical pattern that is recapitulated by stimulation of the appropriate sensory modality. The amygdala receives sensory input from a variety of sources, and in anesthetized animals, neurons in the basolateral amygdala (BLA) show slow rhythmic synchronized activity. Extracellular field potential recordings show that these oscillations are synchronized with sensory cortex and the thalamus, with both the thalamus and cortex leading the BLA. Using whole-cell recording *in vivo* we show that the membrane potential of principal neurons spontaneously oscillates between up- and down-states. Footshock and auditory stimulation delivered during down-states evokes an up-state that fully recapitulates those occurring spontaneously. These results suggest that neurons in the BLA receive convergent input from networks of cortical neurons with slow oscillatory activity and that somatosensory and auditory stimulation can trigger activity in these same networks.

## Introduction

Slow rhythmic oscillations are observed in the cortex during slow-wave sleep, quiet wakefulness [1,2] and under anesthesia [3,4]. At the cellular level, this activity is seen as regular transitions of the membrane potential between depolarized and hyperpolarized states, described as up-states and down-states, respectively [3,5]. Up-states show a pattern of activity that is precisely timed from their onset, and this pattern is recapitulated by sensory stimulation [6]. These slow oscillations are generated in the cortex, but are modulated by thalamic input [7,8], and are thought to play an important role in memory consolidation by transiently grouping the activity of neuronal assemblies [9].

Sensory stimulation-evoked up-states in primary cortical areas are related to the sensory modality stimulated. For example, whisker stimulation evokes up-states in barrel cortex [10]. However, how integrative brain regions that receive and process information from multiple sensory cortices, and display slow oscillations, respond to different sensory stimuli is not well understood. We have previously shown that, under urethane anesthesia, neurons in the basolateral amygdala (BLA) display spontaneous up and down-state transitions [11] that can also be initiated by somatosensory stimulation (footshocks). The BLA receives afferent input from multiple regions of the cortex [12,13], but contradictory evidence exists regarding the functional integration of sensory inputs at the single cell level [14,15]. However, these latter studies used extracellular recordings to study neuronal responses to sensory stimulation, and it is conceivable that some of the evoked activity remained subthreshold and thus undetected by this method. We combined somatosensory and auditory stimulation with *in vivo* whole-cell recordings of principal neurons and interneurons within the BLA to ask whether somato-sensory and auditory inputs converge on single neurons. Our results show that single neurons in the BLA receive convergent input from multiple sensory areas and suggest that slow-wave oscillations in individual BLA neurons are driven by multiple areas of the sensory cortex.

## Methods

Data were obtained from 34 Wistar rats (P16–P25; 40±10 g) for whole-cell recordings and 10 animals for local field potential (LFP) recordings. Animals were housed under standard laboratory conditions with a 12-h light/dark cycle (lights on at 0700 hours), with food and water available *ad libitum*. In all procedures, the care and experimental use of animals was in accordance with protocols approved by the University of Queensland Animal Ethics Committee.

### Surgery

Animals were anesthetized with either intraperitoneal urethane (2 mg/kg, n = 24 for whole-cell recording, n = 10 for LFP recording) or isoflurane (1.5–2% in air, n = 10). Once a sufficient level of anesthesia was obtained, animals were mounted in a stereotaxic frame, and their body temperature was maintained at 37°C. Following exposure of the skull surface, a hole was drilled above the area of interest, and the electrode was lowered to the following coordinates (using bregma as a reference): BLA (AP: -2.6mm, ML: 4.3 mm D: 6.2–6.8mm), primary auditory cortex (A1; AP: -3.6mm, ML: 6mm, D: 1.3mm), primary somatosensory cortex (S1; AP: -1mm, ML: 6 mm, D: 5mm) or posterior thalamus (PoT; AP: -5mm, ML: 3mm, D: 5mm). In LFP studies, we did not record from more than 2 target regions simultaneously. For ipsilateral prefontal cortex stimulation a bipolar concentric electrode was implanted 2.5 mm anterior, 0.5 mm lateral and 3 mm ventral to bregma.

### Evoked activity

Footshocks (5–8 mA, 1 ms) were delivered using an isolated current generator (Digitimer) via two 25-gauge needles inserted into the footpad contralateral to the side of the BLA recording. Auditory stimulation was delivered using a hollow ear bar, and sounds (white-noise or pure tones 3–8 kHz, 75±3 dB, duration: 100–500 ms) were delivered contralateral to the recording site. The amplitude and frequency of the auditory stimuli were varied until a response was evoked, at which point those parameters were kept constant for the remainder of the experiment.

### Electrophysiology

Recording pipettes (shank length of 7.5 mm) were fabricated from borosilicate glass using a Sutter P-87, 3-stage pipette puller. For voltage-clamp recordings, electrodes were filled with a cesium based internal solution containing (in mM): 120 CsMeSO_4_, 20 TEACl, 10 HEPES, 2 Mg_2_ATP, 0.3 Na_3_GTP, 0.1 spermine, 10 phosphocreatine, 5 EGTA, 2 CsBAPTA, 5 QX-314, and 0.3% neurobiotin; (pH 7.3; osmolarity, 310 mOsm). For current-clamp recordings, pipettes were filled with a potassium based internal solution containing (in mM): 135 KMeSO_4_, 7 NaCl, 10 HEPES, 2 Mg_2_ATP, 0.3 Na_3_GTP, 0.3 EGTA, and 0.3% neurobiotin (pH 7.3; osmolarity, 290–300 mOsm). Pipettes had a series resistance of 5–10 MΩ and varied between 20 and 50 MΩ during recordings. Recordings with series resistance higher than this were discarded from the final analysis. Signals were amplified using a Multiclamp 700B amplifier, filtered at 5 kHz, and digitized at 10 kHz using an Instrutech ITC-18 board. For LFP recordings, pipettes were filled with a solution of 3M saline and signals were amplified as described above but band pass filtered between 0.1 and 500 Hz. For all recordings a ground electrode was placed under the scalp.

Data acquisition and subsequent analysis were performed using Axograph X (Axograph Scientific). After completion of the recordings, animals were transcardially perfused with 2% sodium nitrite solution (in 0.1 M phosphate buffer, pH 7.4), followed by 50 ml of 4% formaldehyde (in 0.1 M phosphate buffer, pH 7.4). Brains were removed and post-fixed overnight in 4% formaldehyde at 4°C. Serial, coronal forebrain (100 μm) sections were cut using a freezing microtome and processed using DAB-based immunohistochemistry to recover the recorded cell [11]. All cells used for this study were confirmed to be located in the BLA.

### Data analysis

Individual traces (n = 10–15) of spontaneous or evoked events recorded in current clamp were analyzed using Axograph and their duration, power (area under the trace) and peak amplitude measured. For voltage-clamp experiments, we followed the protocol and analysis described by Windels et al. (2010). Briefly, individual traces (n = 8–10) of spontaneous or evoked currents occurring at -50 mV (excitatory current) and +20 mV (inhibitory current) were used to calculate the duration, power and peak amplitude for each current. The latency to up-state onset was measured as the time from the stimulus artifact to when the trace deflection attained 5% of the initial peak. Power was calculated as the area under the curve.

### Principal component analysis

Principal component (PC) analysis was conducted on all spontaneous and evoked events from current-clamp or voltage-clamp recordings using custom routines in Matlab. Each event was transformed into a point in PC space, and for each PC dimension, the coordinates of all points (i.e. events) in a recording were z-scored. A two-sample Kolmogorov-Smirnov (K-S) test was used to detect any difference between spontaneous and evoked events in each PC dimension. As the K-S test is sensitive to a difference in overall distribution, but not to individual outliers, individual triggered events outside the confidence limit in any PC dimension (see below for details of confidence limit calculation) were considered different from the spontaneous events in that recording. The process was repeated for the first 50 PCs in each recording; we report the variance explained for the PCs whenever a difference was identified between spontaneous and evoked events (see S1 Fig for details).

The confidence limit (±*X*) was calculated assuming that all events (i.e. point coordinates) were normally distributed in each dimension. First, the total number of PC dimension tests (*d*) for each recording was calculated–this was simply the number of PC dimensions (50) multiplied by the number of triggered events in that recording. We then set the probability *P* at 0.01 with Bonferroni correction for the number of tests, ie *P* = 0.01/(2d). We calculated the z-scored confidence level X using the Matlab *norminv* command–that is, the inverse of the cumulative normal distribution function with zero mean and unit standard deviation (i.e. *M* = 0, *S* = 1), such that:

$$\text{P}=\int_{-\infty }^{X}\frac{1}{\sqrt{2\pi \times S}}\;e^{-\frac{{\left(t-M\right)}^{2}}{{2S}^{2}}dt}$$

If any test for a recording was observed to be outside X in any dimension, we inferred that the corresponding triggered event was separable from the spontaneous event.

### Statistics

Results obtained with a common anesthetic were tested for significant difference using Friedman’s test with Dunn’s multiple comparisons correction; the Wilcoxon rank sum test was used for comparisons between anesthetics. Results are presented as mean ± SD unless otherwise specified and n values indicate the number of recordings. Because sensory stimulation recapitulates the overall shape of the spontaneous events (see below) we only compared spontaneous events between anesthetics.

## Results and Discussion

**Fig 1:**
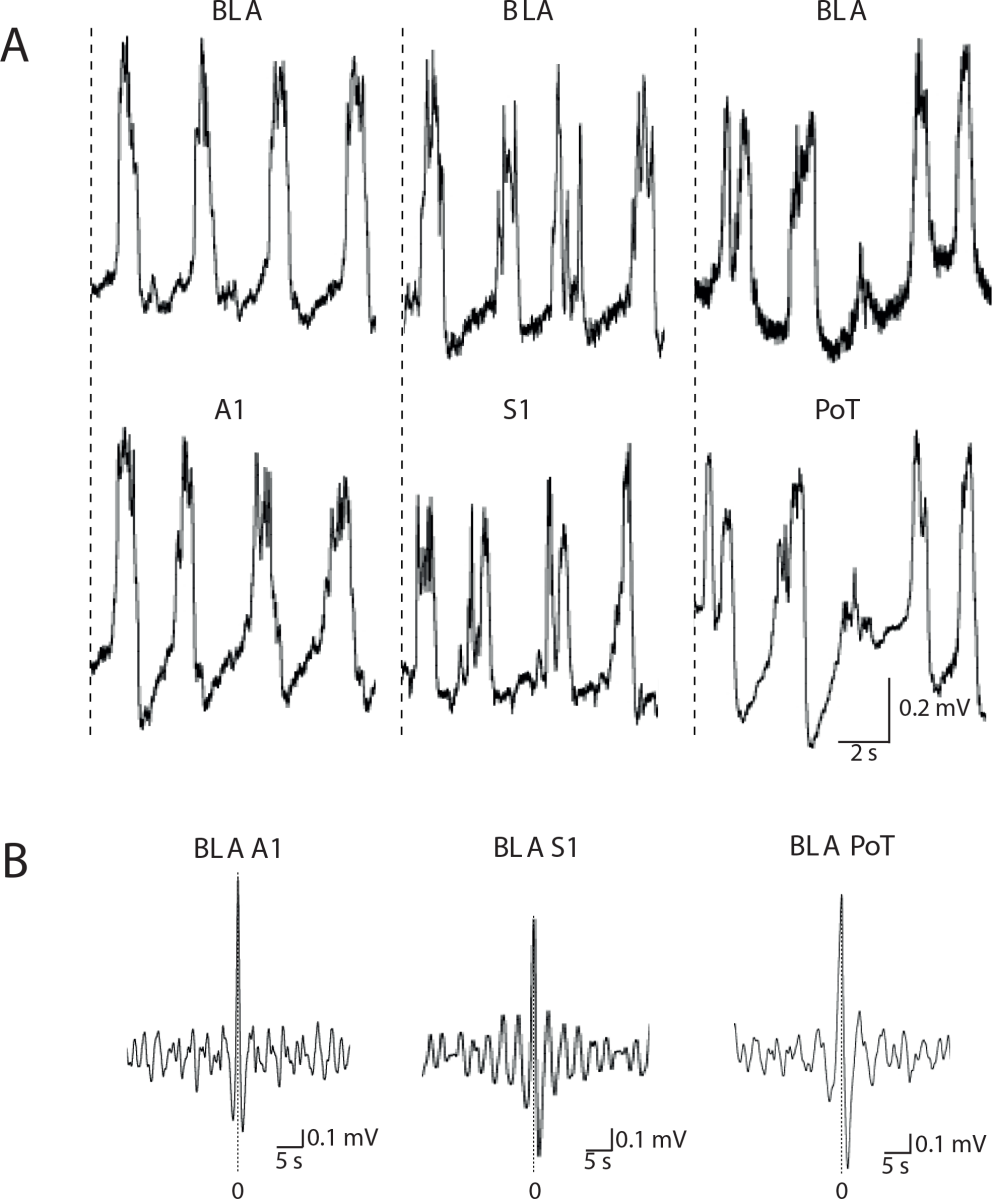
Oscillations in the basolateral amygdala (BLA) are synchronized with those in primary auditory cortex (A1), primary sensory cortex (S1) and posterior thalamus (PoT). Illustrated are local field potential (LFP) recordings of spontaneous oscillations in an urethane anesthetized animal in the BLA, cortex and thalamus. Shown is a 10 s trace of simultaneous recording of LFP in the BLA (A, top row) and A1, S1 or PoT (A, lower row). (B) Cross-correlograms of a 30 s recording from sites presented in (A), centred on the peak at zero (0) lag.

Under urethane anesthesia, neurons in the BLA [11], cortex and thalamus [16] display slow oscillations in membrane potential. As the BLA receives strong projections from most cortical regions [13], we have previously suggested that the slow oscillations in BLA neurons could be driven by excitatory cortical input [11]. To test this hypothesis, we conducted simultaneous LFP recordings from the BLA (n = 11) and the cortex (areas A1 [n = 3], S1 [n = 4]) or thalamus (PoT [n = 4]). Spontaneous oscillations were apparent in the LFP in all regions (Fig 1A) with the following mean frequencies: BLA, 0.307 ± 0.062 Hz; A1, 0.376 ± 0.098Hz; S1, 0.329 ± 0.059 Hz and PoT, 0.262 ± 0.050 Hz. The LFPs recorded in BLA are most probably generated by temporally synchronized input to this region, as no consistent spatial orientation of the dendritic fields has been described [17]. As compared to the BLA, the oscillation frequency was not significantly different (Wilcoxon rank test for paired data) in cortex or thalamus (BLA and Cortex: p = 1, n = 7; BLA and thalamus: p = 0.25, n = 4). Cross-correlational analysis revealed tightly correlated activity between the BLA and the cortex or thalamus (Fig 1B), with lag-times of 9.00 ± 7.23 ms and 43.98 ± 11.34 ms for Cortex (A1, S1) and PoT respectively. Thus, cortical and thalamic activity both lead the BLA, a result consistent with previous reports from cortical and subcortical recordings [18, 19, 20], suggesting that neocortical activity drives BLA oscillations.

**Fig 2:**
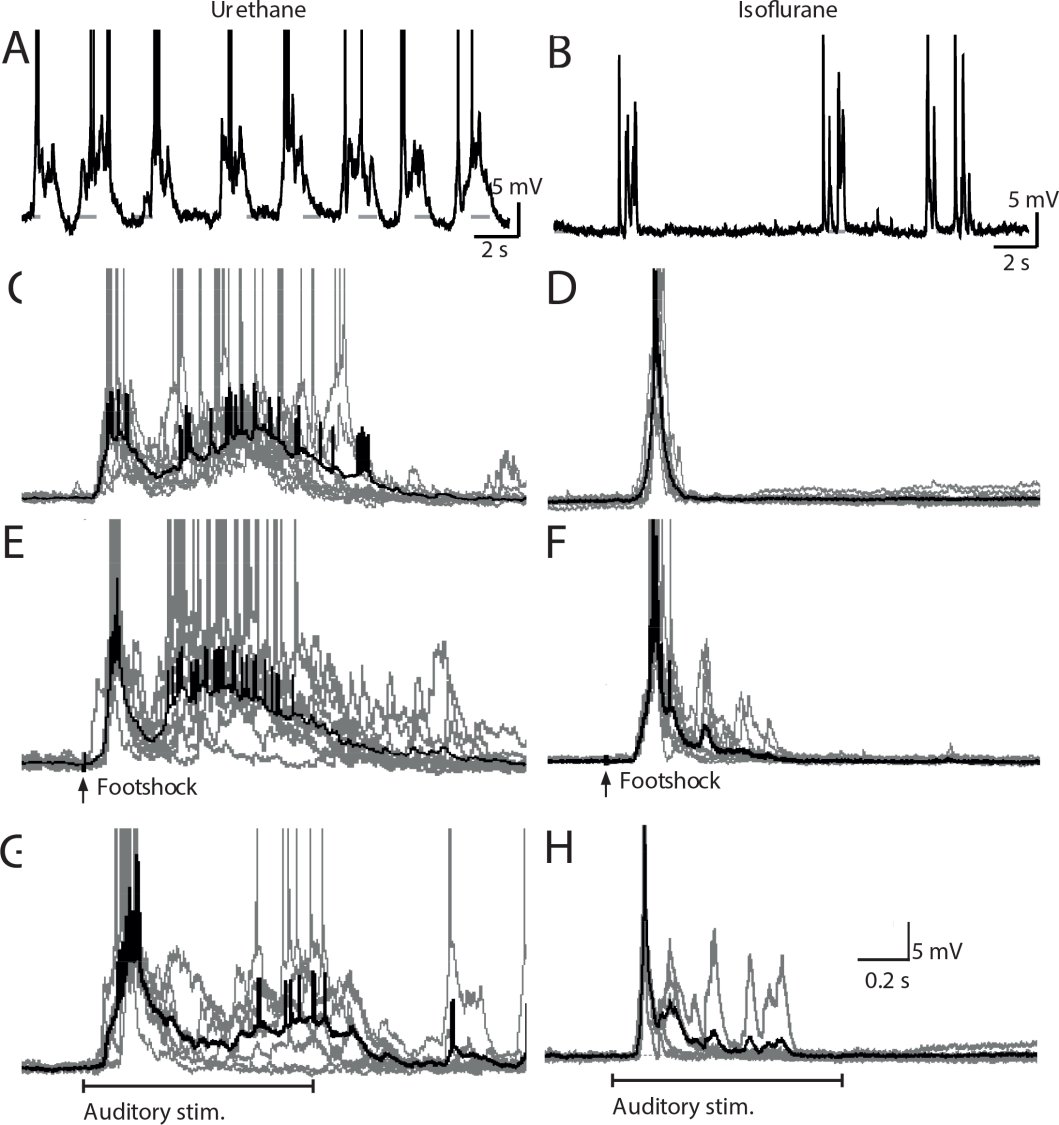
Urethane and isoflurane have different effects on neural activity recorded in principal neurons of the BLA. Whole-cell current-clamp recordings from BLA principal neurons in urethane (A) and isoflurane-anesthetized (B) rats. Spontaneous up-states with urethane (C) and isoflurane (D) anaesthesia are shown on an expanded time scale. (E-G), Footshock and auditory stimulation delivered in the down-state evoke responses similar to spontaneously occurring events in both urethane (E, G) and isoflurane (F, H)-anesthetized animals. Individual traces are represented in gray and average traces in black, horizontal gray dotted line represent the average voltage of the quiescent state in the period preceding event onset (C-H).

We next sought to establish the impact of sensory stimulation on membrane oscillations of individual neurons in the BLA. Whole-cell recordings were obtained from principal neurons in the BLA, *in vivo*. Most recordings were made in animals anesthetized with urethane (17 cells recorded in 15 animals). However, as the properties of neurons can differ under different anesthetics [21], some recordings were also obtained under isoflurane (10 cells recorded in 10 animals). Resting membrane potential (V_m_) and input resistance (R_in_) under the two anesthetics were similar (V_m_: urethane, -57.47 ± 5.91 mV compared to isoflurane, -55.97 ± 8.25 mV, p>0.99; R_in_: urethane, 64.72 ± 21.08 MΩ compared to isoflurane, 62.21 ± 21.78 MΩ, p>0.99), and the firing properties in response to current injection were also similar to those previously described *in vitro* [22] and *in vivo* [11]. As reported previously, all neurons in urethane-anesthetized animals displayed a low frequency (0.34 ± 0.17 Hz) oscillation in membrane potential between the resting and a depolarized state (Fig 2A) [11,23], similar to those reported in cortical neurons under similar conditions [24]. In contrast, under isoflurane anesthesia the membrane potential was more stable with no consistent oscillatory activity. However, sporadic bursts of activity were present (Fig 2B), consistent with the low basal activity of principal neurons reported during single unit recordings *in vivo* [25]. The mean duration and power (integrated area under curve) of spontaneous events was greater under urethane anesthesia than isoflurane (Fig 2C and 2D, Table 1, p<0.001), but the peak amplitude of spontaneous events did not differ significantly between the two anesthetics (p = 0.22). In the remainder of this article, the quiescent hyperpolarized state will be referred to as the down-state and the depolarized state will be referred to as the up-state.

**Table 1:** Summary table of current-clamp results. Peak amplitude, area under the curve, duration and latency for spontaneous and evoked events recorded in BLA of urethane- and isoflurane-anesthetized animals. Results are presented as mean ± SD.

		Peak amp. (mV)	Area (mV.s)	Duration (ms)	Latency (ms)
Isoflurane	Spontaneous	11.81±4.31	2.25±1.32	445.93±242.18	n/a
	Auditory stimulation	8.27±4.77	1.85±1.12	452.38±245.39	121.5±8.21
	Footshock	10.67±4.13	1.93±1.37	476.21±245.03	102.30±37.04
Urethane	Spontaneous	12.07±3.54	10.29±3.97	1358.09±229.42	n/a
	Auditory stimulation	11.53±4.34	8.89±4.84	1337.05±229.42	66.99±35.18
	Footshock	11.58±4.89	7.77±3.67	1149.02±347.55	79.06±28.22

Cortical areas are generally silent under isoflurane anesthesia [26], but remain responsive to sensory stimulation. In BLA principal neurons, auditory stimulation or footshock delivered during down-states evoked an up-state, regardless of the type of anesthetic (Fig 2E–2H). However, the latency from the stimulation to the onset of the evoked response was significantly shorter under urethane, compared with isoflurane-anesthetized animals (Table 1, p<0.05). This result is in agreement with the longer latency of sensory-evoked activity reported in cortical recordings under isoflurane anesthesia [27]. Overall, with either anesthetic, the latency from stimulation to evoked responses (66–121 ms; Table 1) is consistent with the notion that activation of the cortico-thalamic networks [28] drives oscillatory activity in BLA neurons. As previously described in cortical neurons [29], neither auditory stimuli nor footshock had any detectable effect when delivered during the up-state.

We reported previously that a footshock delivered during the down-state resets the phase of the slow membrane potential oscillation [11]. In the current study, under urethane anesthesia, the time from the onset of an evoked up-state to the next spontaneous up-state was 2.75±0.40 s (n = 8) following footshock, and 2.93±0.26 s (n = 7) for auditory stimulation. These values are not significantly different to the mean inter up-state interval during spontaneous oscillations (2.92±0.72 s; n = 11, p = 0.99). Thus, as with footshock [11], auditory stimulation in the down-state also resets the phase of the membrane potential oscillation in BLA neurons.

Footshock and auditory stimulation delivered in the down-state evoked responses that closely resembled spontaneous events (Fig 2E–2H, Table 1). To quantitatively evaluate the similarity between spontaneous and evoked up-states, we used principal component (PC) analysis, a method that does not require any assumptions about the nature of potential differences. A Kolmorgorov-Smirnov (K-S) test on each of the first 50 PC dimensions of the upstates of each recording (n = 18 recordings, giving 900 K-S tests in total) revealed that only 1.3% (12/900) showed significant differences for the evoked vs spontaneous upstates. However, as the K-S test is sensitive to a difference in overall distribution but not to individual outliers, we next tested each individual triggered upstate (n = 508 triggered upstates in the 18 recordings) on each PC dimension to determine whether it lay outside the expected range of spontaneous events. This analysis revealed that only 6.7% (34/508) of triggered events were distinguishable from spontaneous events. Moreover, only 3.6% (32/900) of the PCs were different for the distinguishable events (see Methods) and each PC represented, on average, only 0.40% of the variance, indicating that these PCs were only minor contributors to the overall variance in the triggered upstate waveforms. Thus, similar to results in auditory cortex [29], we conclude that sensory evoked up-states in the BLA are almost identical to spontaneously occurring up-states.

**Fig 3:**
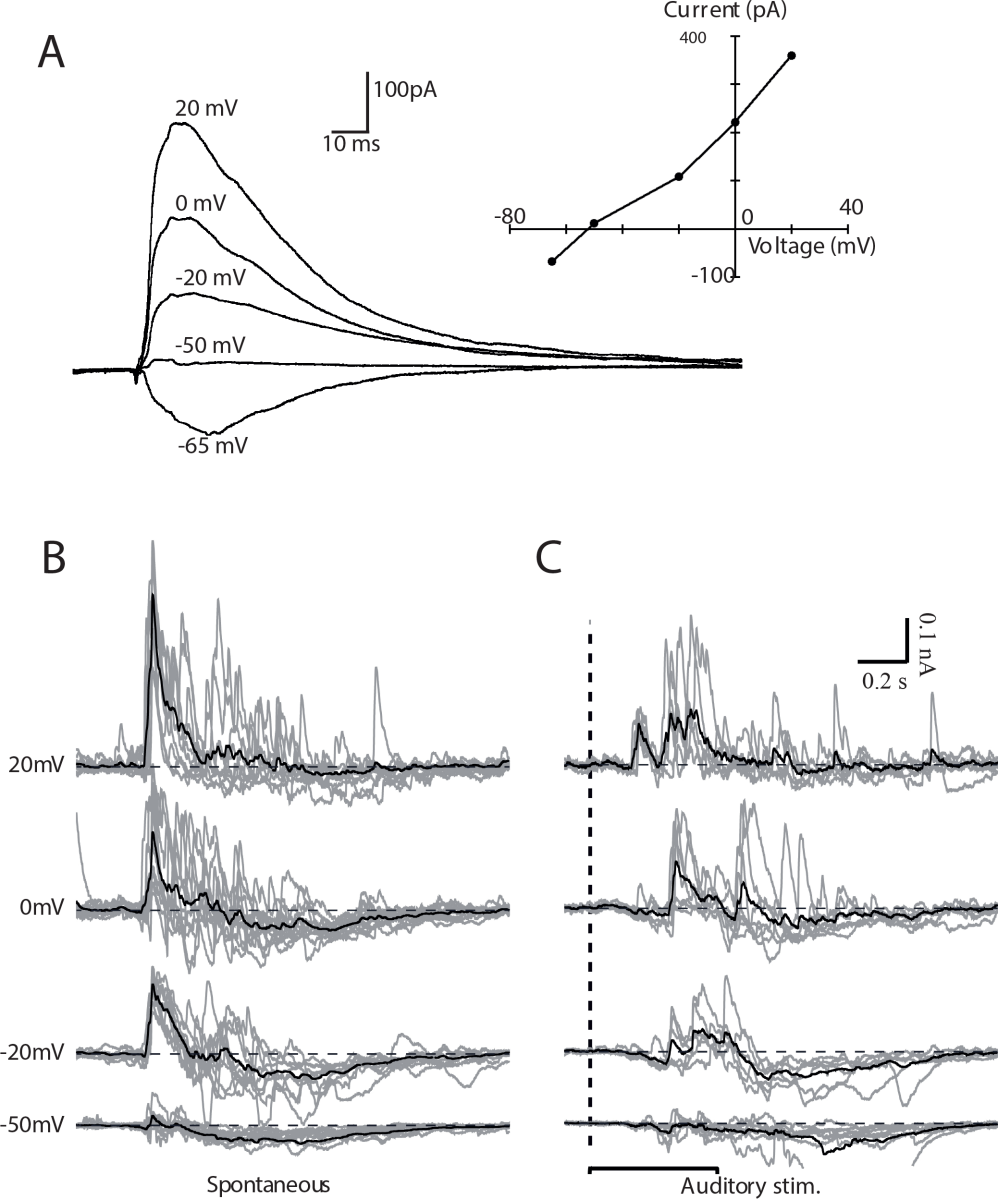
Auditory stimulation evokes similar synaptic currents to those that drive spontaneous events. Whole cell recording was obtained from neurons in the rat BLA *in vivo*. Synaptic currents evoked by prefrontal cortex stimulation were recorded from a principal neuron in voltage-clamp at the indicated holding membrane potentials (A). Current-voltage relationship (A, insert) obtained from recordings of synaptic currents described in A. B, C Voltage-clamp recordings of spontaneous (B) and auditory evoked activity (C) at the indicated membrane potentials. Individual traces are represented in gray and average traces in black, horizontal black dotted line represent the average voltage of the quiescent state in the period preceding event onset (B-C).

We have previously shown that the synaptic input driving footshock-evoked up-states is dominated by an early, short duration, inhibitory component [23]. To compare the currents evoked by auditory stimulation and footshock, neurons were voltage-clamped in urethane-anesthetized animals (5 cells in 3 animals). To assess the fidelity of our voltage-clamp *in vivo*, we first tested the response to stimulation of the medial prefrontal cortex, which has strong direct projections to the BLA [30, 31]. Prefrontal cortex stimulation evoked a robust synaptic input that was dominated by an inhibitory component [23], which may in part result from feedback inhibition activated by backfiring of principal neurons in the BLA that project to the prefrontal cortex [32]. The current-voltage relationship of the prefrontal cortex evoked input was linear, with a measured reversal potential of the inhibitory input, of -60 mV (Fig 3A). These results show that despite a series resistance that ranged from 20–50 MΩ, we could reasonably voltage-clamp inhibitory inputs onto BLA neurons *in vivo*.

Synaptic currents occurring either spontaneously or in response to sensory stimulations were recorded over a range of membrane potentials (Fig 3B and 3C). Excitatory inputs were quantified at a holding potential of -50 mV, near the reversal potential for inhibitory chloride currents, and inhibitory inputs were quantified at a holding potential of +20 mV, near the reversal potential for ionotropic glutamate receptors [23]. Subsequent analysis revealed that the synaptic currents driving spontaneous and evoked up-states were not significantly different (Table 2, within cells comparisons, Friedman’s test, p>0.99). Using PC analysis we found that for excitatory events, differences between spontaneous and evoked excitatory events were present in 10% of the PCs tested, representing only 0.36% of the variance. For inhibitory events, differences were present in 8% of the PCs, representing 0.53% of the variance. Together, these results demonstrate that both somatosensory (footshock) and auditory stimulation trigger excitatory and inhibitory synaptic currents in BLA neurons that are very similar to those observed during the spontaneous oscillatory events displayed by these neurons.

**Table 2:** Summary table of voltage-clamp results. Peak amplitude, area under the curve, duration and latency for excitatory and inhibitory currents recorded for spontaneous and evoked events in BLA of urethane-anesthetized animals. Results are presented as mean ± SD.

		Peak amp. (pA)	Area (pA.s)	Duration (ms)	Latency (ms)
Inhibition	Spontaneous	260.68±296.62	111.22±184.74	11292.04±335.42	n/a
	Footshock	281.92±298.16	122.97±200.59	1288.23±319.76	110.06±13.49
	Auditory Stimulation	72.25±104.90	52.20±121.20	1383.96+301. 21	196.40±56.48
Excitation	Spontaneous	60.12±84.88	54.58±39.79	1280.87±398.55	n/a
	Footshock	56.66±69.00	61.01±49.66	1257.16±234.23	112.85±19.10
	Auditory Stimulation	62.54±60.41	90.78±71.61	1434.43±285.44	166.21±53.64

Every neuron tested with both footshock and auditory stimulation responded to both stimuli (n = 22). This result is consistent with anatomical and functional studies showing that neurons from the dorsal region of the lateral amygdala receive inputs from auditory and somatosensory cortices [33,34]. However, unlike these early studies we observed the same convergence throughout the dorso-ventral extent of the BLA. It is conceivable that this difference comes from the methods used to detect responses to sensory stimuli. Our whole-cell recording method allows the detection of subthreshold activity while others used action potential firing as a marker for responses to sensory stimuli [14,15].

**Fig 4:**
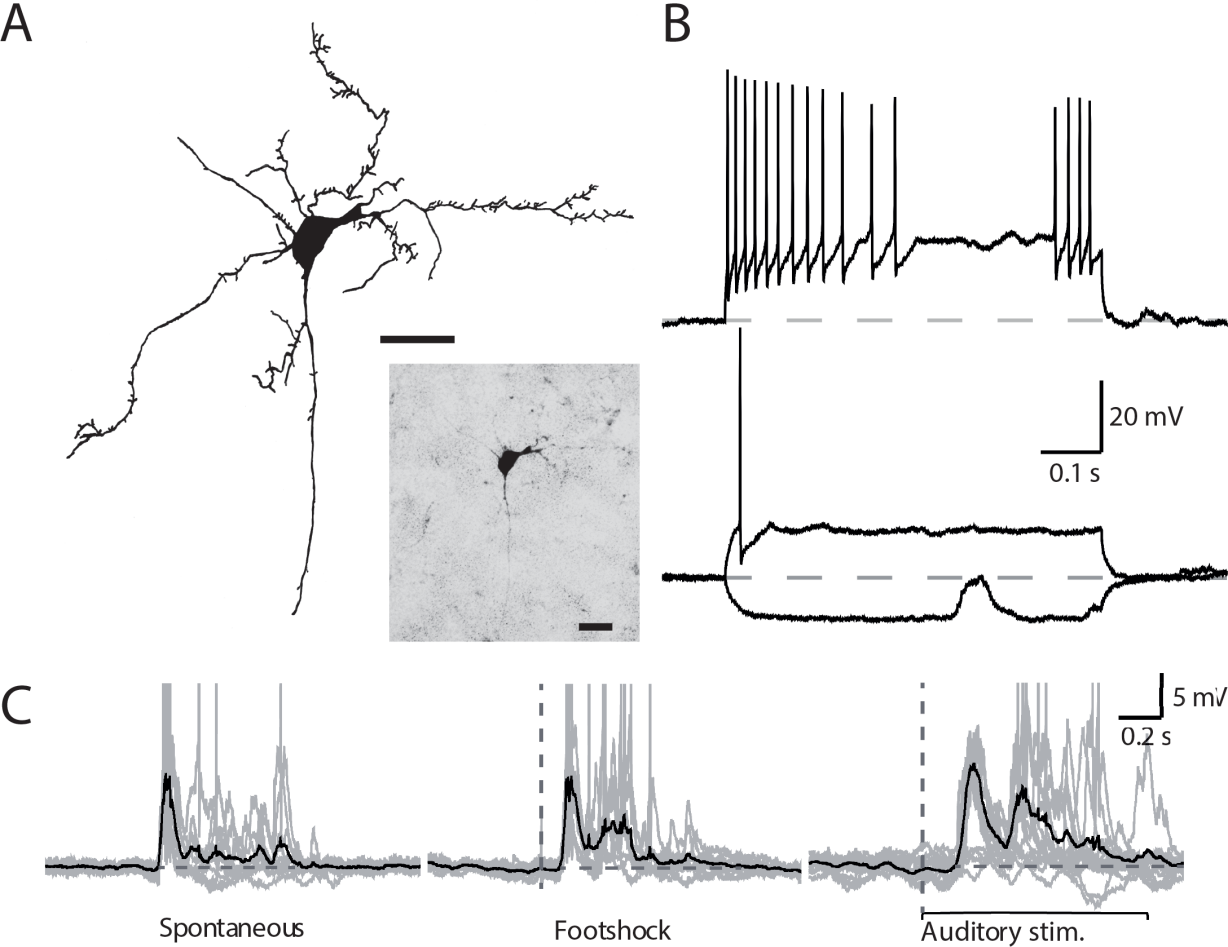
Spontaneous and evoked activity recorded from BLA interneurons in isoflurane-anesthetized animals. A, Camera lucida drawing of the BLA interneuron shown in photomicrograph (insert) recorded under isoflurane anesthesia and filled with neurobiotin. Scale bar: 20μm. B, Firing properties of cell shown in A in response to 600 ms current injections. Interneurons also show spontaneous events that are recapitulated by footshock and auditory stimulation. Shown are average spontaneous events (C, left) and footshock (C, middle) and auditory (C, right) evoked responses in one neuron (individual traces are represented in gray). Horizontal gray dotted line represents the average voltage of the quiescent state in the period preceding event onset.

Whole-cell recording in the amygdala *in vivo* requires a ‘blind patch’ approach. Since we were not able to target a specific cell population, the sample of neurons recorded in this study most probably reflects the overall proportions of projection cells (85%) and putative interneurons (15%) found in this brain region [35, 36]. Despite their scarcity, we were able to record from 3 interneurons (in 3 animals). These cells had aspiny dendrites and fast action potentials (AP half width 0.58 ± 0.031 ms; Fig 4A), with firing properties that were not different from those reported in slice preparations (Fig 4B; see [37] for review). Interneurons also displayed spontaneous and evoked activity which closely matched that seen in principal neurons (Fig 4C). However, following footshock delivery the latency to the peak response in interneurons was slightly shorter (30.65±16.89 ms) as compared to that in principal neurons (49.60±27.78 ms, p<0.001). In contrast, following auditory stimulation the latency to the peak response in interneurons was significantly larger (152.37±20.12 ms) than that seen in pyramidal neurons (122.617± 23.12 ms, p<0.001). The reasons for these differences are not clear, but may reflect differences in the innervation of interneurons by afferents carrying footshock and auditory information.

Previous *in vivo* studies have shown that sensory input can drive neurons in primary sensory cortex into an up-state [1]. Furthermore, in cortico-thalamic slices, thalamic stimulation was shown to fully reproduce spontaneously occurring up-states in layer 4 neurons [38]. On a larger scale, spontaneously active neuronal assemblies can also be re-activated by sensory stimulation in anesthetized animals [39]. The BLA receives input from both the auditory and sensory cortices [13], and our results suggest that the large spontaneous and evoked up-states observed in the BLA are cortically driven. This similarity in response of BLA neurons to distinct sensory stimuli is in line with the broad cortical input that this region receives [12], but how somatosensory and auditory inputs, which have entirely different connections, drive near identical responses in the BLA is not obvious.

Using cortical LFP recording in isoflurane anesthetized animals Hudetz et al. reported that spontaneous activity could be recapitulated by visual stimuli [40]. Moreover, in the Hudetz et al. study, while a short latency response was first observed in V1 it was followed by responses in somatosensory, motor cortex and ultimately in olfactory bulb the resembled the spontaneously occurring event. In addition, other sensory modalities are able to evoke the same type of response [41]. We therefore propose that the heteromodal response to a unimodal sensory stimulus at the cortical level will also be recorded in the BLA, and explains the ability of both footshock and auditory stimuli to recapitulate spontaneously occurring bouts of activity in BLA neurons.

The fact that this similarity is seen under either urethane or isoflurane suggests that it is likely to be due to the nature of cortical connections to the BLA. The broad connectivity of the BLA and the similarity of its response to diverse sensory stimulation is consistent with the suggestion that it should be viewed as a ‘hub’ for cortical network activity [42].

## Supporting Information

S1 Fig (A) The overall shape of evoked up-states is similar to spontaneous up-states in principal component space. The first three PCs are plotted against each other for the data from one illustrative recording that had the largest difference between evoked (grey dots) and spontaneous up-states (black dots). (B) Cumulative explained variance for the first 50 principal components for the up-states shown in the clusters from S1A. The 50% and 80% explained variance levels are marked. (C) Distribution of PC1 values for spontaneous (black) and evoked events (grey). (D) Median upstate overlaid on the first 5 principal components for the up-states shown in the clusters from S1A.
(EPS)
